# Dobutamine stress echocardiography in healthy adult male rats

**DOI:** 10.1186/1476-7120-3-34

**Published:** 2005-10-26

**Authors:** Eric Plante, Dominic Lachance, Marie-Claude Drolet, Élise Roussel, Jacques Couet, Marie Arsenault

**Affiliations:** 1 Groupe de Recherche sur les Valvulopathies, Centre de Recherche Hôpital Laval, Institut de cardiologie de Québec, Université Laval, Québec, Canada

**Keywords:** Dobutamine, stress echocardiography, rat, animal model, stress

## Abstract

**Background:**

Dobutamine stress echocardiography is used to investigate a wide variety of heart diseases in humans. Dobutamine stress echocardiography has also been used in animal models of heart disease despite the facts that the normal response of healthy rat hearts to this type of pharmacological stress testing is unknown. This study was performed to assess this normal response.

**Methods:**

15 normal adult male Wistar rats were evaluated. Increasing doses of dobutamine were infused intravenously under continuous imaging of the heart by a 12 MHz ultrasound probe.

**Results:**

Dobutamine stress echocardiography reduced gradually LV diastolic and systolic dimensions. Ejection fraction increased by a mean of +24% vs. baseline. Heart rate increased progressively without reaching a plateau. Changes in LV dimensions and ejection fraction reached a plateau after a mean of 4 minutes at a constant infusion rate.

**Conclusion:**

DSE can be easily performed in rats. The normal response is an increase in heart rate and ejection fraction and a decrease in LV dimensions. A plateau in echocardiographic measurements is obtained after 4 minutes of a constant infusion rate in most animals.

## Introduction

Dobutamine stress echocardiography (DSE) is commonly used in clinical practice to investigate patients with a wide variety of cardiovascular diseases. DSE can be used for many purposes such as to seek myocardial ischemia, evaluate valvular diseases, measure myocardial viability and assess myocardial contractile reserve [1-8]. Improvements in cardiac ultrasound imaging devices in recent years have allowed investigators to use echocardiography in small animals with cardiac diseases such as rats and mice and obtain images of very good quality. Consequently, research groups are now routinely using cardiac ultrasound imaging not only to investigate large animals (such as dogs and pigs) but also small rodents. Dobutamine stress imaging has also emerged in those small animals. Investigators working with small animal models of cardiac diseases have been using dobutamine stimulation mostly to assess myocardial contractile reserve. Accurate interpretation of a stress test requires that the response of normal subjects to that test is well documented. However, there is currently no available data on the normal response to dobutamine stimulation of the hearts of rats and we do not know if they respond to DSE as humans do. Therefore, the present study was designed to assess the normal response of rat hearts to DSE.

## Methods

### Animals

15 adult male Wistar rats (400–450 g) were investigated. This protocol was approved by the Université Laval's animal protection committee and was consistent with the recommendations of the Canadian Council on animal care.

### Dobutamine Stress Echocardiography

The animals were sedated with isoflurane (2.5%, inhaled). The femoral vein was dissected and cannulated for the intravenous dobutamine infusion. The femoral artery was also cannulated to measure arterial blood pressure. The thorax was shaved and the animal was put on a dorsal decubitus position. Imaging of the heart was done using a 12 MHz ultrasound probe and a Sonos 5500 echographer (Philips Medical Imaging, Andover, MA). Imaging depth was adjusted for each animal to obtain the largest possible 2D image including the whole left ventricle in the imaging sector. Harmonic imaging was used for all the studies with optimal adjustment of gain to ensure the best quality of images and adequate delineation of endocardial borders. Images were stored on magneto-optical disks for off-line analysis. Baseline measurements were obtained under isofurane anesthesia before the beginning of dobutamine infusion and continuous imaging was done during the dobutamine test. Results were compiled at every minute during the infusion.

### 2 different dobutamine infusion protocols were tested

#### Protocol #1

Dobutamine was infused intravenously at a constant rate of 10 μg/kg/min until a plateau was reached in the left ventricular (LV) ejection fraction for at least 2 minutes (n = 10 rats). Similar doses of dobutamine infusion has been used by others to evaluate myocardial contractile in rats [9].

#### Protocol #2

Progressively increasing doses of dobutamine (5, 10 and 20 μg/kg/min) were infused in this protocol (n = 5 rats). Each dose was infused for 4 minutes before imaging and increasing to the next step. This protocol of progressively increasing doses is closer to DSE protocols used in clinical practice and has been used by other investigators [10] using other imaging techniques.

Left ventricular dimensions and septal and posterior wall thicknesses were obtained in the parasternal long axis view using M-mode imaging. Relative wall thickness (RWT) was calculated as the ratio of the sum of the wall thicknesses (septal and posterior) to the end-diastolic diameter. Ejection fraction was calculated using the formula of Quinones et al. [11].

### Statistical analysis

Results are presented as mean ± SEM unless specified otherwise. Paired Student t tests were used to compare the results at baseline with those at the end of dobutamine infusion. One-way analysis of variance was performed to compare serial data. Statistical significance was set at a p value of 0.05 or less using post-hoc Tukey's test. Data and statistical analysis were performed using GraphPad Prism version 4.02 for Windows, GraphPad Software (San Diego California USA).

## Results

### Feasibility

Dobutamine stress echocardiography was easily performed in all animals and well tolerated. All animals survived the procedure and we did not encounter any complications.

### Hemodynamic response (fig. 1 and 5a)

**Figure 1:**
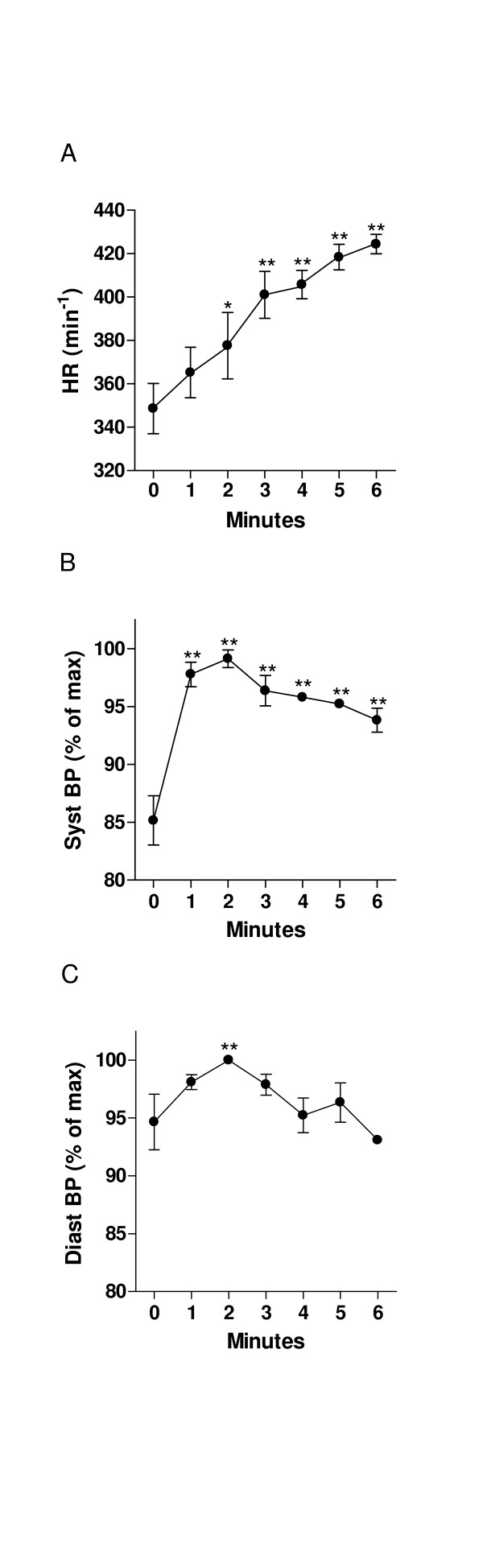
Hemodynamic response to dobutamine infusion in rats (protocol #1). A: heart rate, B: systolic blood pressure (expressed as % of maximal response) and C: diastolic blood pressure (expressed as % of maximal response). *: p < 0.05 vs. baseline. Data is presented are mean ± SEM.

**Figure 5:**
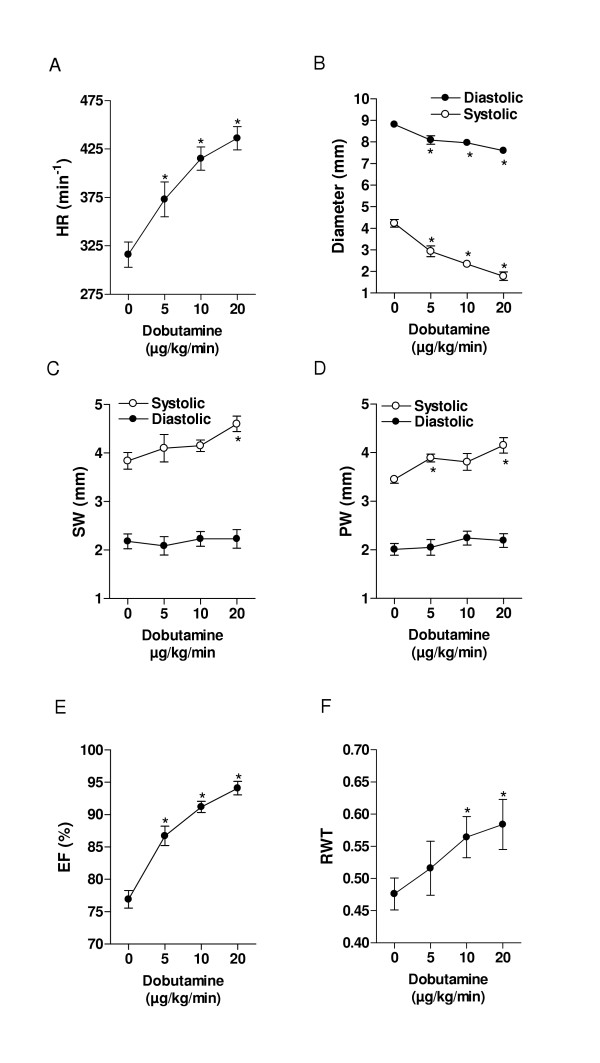
Dose-response effects of dobutamine infusion on the LV dimensions and ejection fraction (protocol #2). A: Heart rate, B LV diameters, C: septal wall, (SW). D posterior wall, (PW) E: Ejection fraction (EF) and F: relative wall thickness (RWT). Closed circles: diastolic measurements; Open circles: systolic measurements*: p > 0.05 vs. baseline. Data are presented as mean ± SEM.

#### Protocol #1

Heart rate and blood pressure response to dobutamine infusion are displayed in figure 1. There was a constant increase in heart rate (Baseline: 349 ± 12 bpm vs. Dobutamine: 424 ± 4 bpm, p < 0.01) in all the animals during the infusion of dobutamine (fig. 1a). Systolic blood pressure sharply increased after only 1 minute of infusion and reached a plateau rapidly after (fig. 1b). We observed a trend towards a mild increase in diastolic blood pressure after 2 minutes of infusion but this did not reach statistical significance and diastolic pressure remained comparable to baseline for the rest of the protocol (fig 1c).

#### Protocol #2

Animals in protocol #2 responded in a similar fashion to protocol #1 with a progressive increase in heart rate with each step (fig 5a), and a plateau in systolic and diastolic pressure (not shown).

### ECG monitoring

ECG was continuously monitored in 5 animals. All animals progressively developed sinus tachycardia (see above) with dobutamine stimulation as expected. We did not notice any ST segment change nor any form of ventricular or supra-ventricular arythmia.

### LV dimensions and ejection fraction (fig. 2, 3, 4)

**Figure 2:**
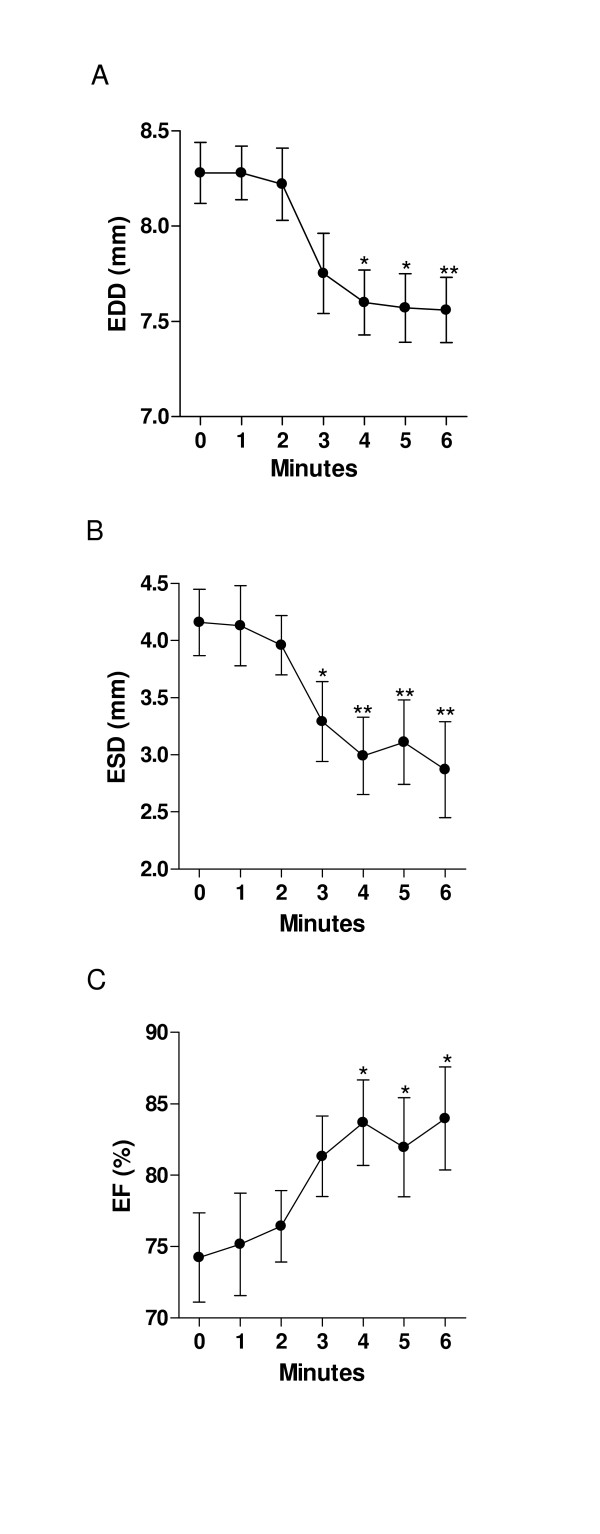
Left ventricular dimensions and ejection fraction during dobutamine infusion (protocol #1). A: End-diastolic diameter B: end-systolic diameter C: ejection fraction. *: p < 0.05 vs. baseline. Data are presented as mean ± SEM.

**Figure 3:**
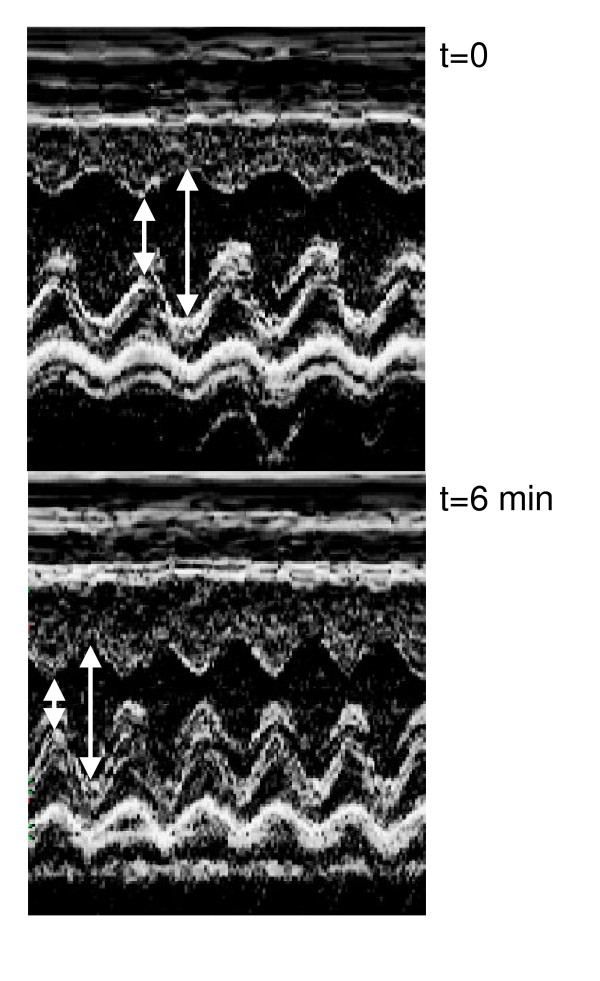
Representative M-mode long axis view of the left ventricle at before (t = 0) and after 6 minutes (t = 6) of dobutamine infusion.

**Figure 4:**
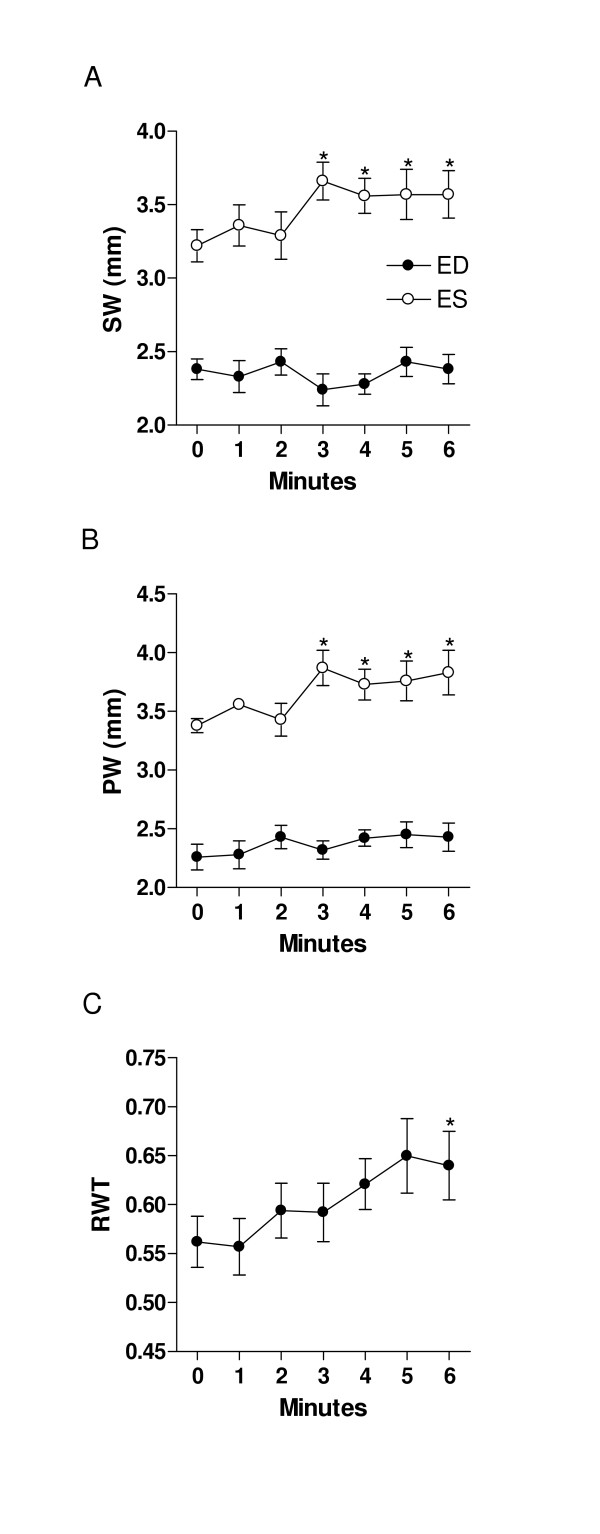
Left ventricular wall thickness and relative wall thickness during dobutamine infusion. A: septal wall (SW), B: posterior wall (PW) and C: relative wall thickness (RWT). Closed circles: diastolic measurements; Open circles: systolic measurements. *: p < 0.05 vs. baseline. Data are presented as mean ± SEM.

#### Protocol #1

Dobutamine infusion reduced LV dimensions as shown in fig 2 and 3. End-diastolic dimensions (fig 2a) progressively decreased compared to baseline, reaching a plateau after 4 minutes (Baseline: 8.5 ± 0.14 mm vs. Dobutamine: 7.9 ± 0.28 mm, p < 0.01). The effects of dobutamine were more prominent on end-systolic dimensions (fig. 2b) with a measured decrease from 4.1 ± 0.24 mm to 2.6 ± 0.27 mm (p < 0.01). As for diastolic dimensions, a plateau was reached in end-systolic dimensions after a mean of 4 minutes of infusion. Ejection fraction behaved similarly with an increase from 74 ± 3.1% at baseline to 84 ± 3.6% with dobutamine (fig. 2c). The left ventricular cavity transiently remodeled in a concentric fashion during the dobutamine infusion as depicted by the progressive increase in relative wall thickness (fig. 4c). Diastolic septal and posterior wall thickness were unaffected by dobutamine whereas systolic thickening increased under dobutamine stimulation as expected under an inotropic stimulus (fig. 4a and 4b). Significant increases in systolic thickening was observed only after a mean of 3 minutes of dobutamine infusion and remained constant afterwards.

#### Protocol #2

Findings in protocol #2 were similar to protocol #1 with a progressive decrease in diastolic and systolic dimensions (fig 5b), increase in systolic thickening (fig 5c–d), increase in ejection fraction (fig 5e) and acute concentric remodeling (fig 5f).

## Discussion

In this study, we demonstrate that dobutamine stress echocardiography is easily feasible in rats and that rat hearts respond to dobutamine infusion in a way that shares strong similarities with humans. Dobutamine infusion in rats is safe, quick and yields important information about the animal's heart response to an adrenergic stress.

Evaluating the response of the animal heart to stress *in vivo *is a quite challenging task. Imaging of the heart on a live, non sedated small animal such as a rat is virtually impossible. Exercise imaging would be even more complex. The explanted heart can be stimulated *ex vivo *in a perfusion setting but the results obtained may not reflect the reactions of the heart in the live animal. Moreover, this type of experimentation does not allow serial evaluations of the heart for obvious reasons.

Dobutamine stress echocardiography allows non-invasive, serial evaluation of the heart in the live animal. DSE is widely used in humans for the evaluation of left ventricular function, contractile reserve, ischemia and viability [1-8]. DSE has proven its usefulness in the investigation of a wide range of cardiac diseases such as cardiomyopathies, valvular diseases, heart failure and ischemic heart disease. The normal response in humans is an increase in heart rate, cardiac output and myocardial contractility. Left ventricular dimensions normally decrease under dobutamine stimulation as a result of the changes in preload and afterload induced by dobutamine. We found similar responses in our normal rats.

Dobutamine stress echocardiography has been used by other investigators using animal models of heart disease but mostly in large animals such as pigs and dogs [12-24]. Little data is available in smaller animals such as mice and rats. When DSE was used in those small animals in previous studies, collected data consisted mostly of fractional shortening or short axis cross sectional area changes or invasive intracardiac measures of dP/dT [25-29]. The global echocardiographic response of the heart to dobutamine stimulation in rats has never been described. Our study is the first to report the normal expected dynamic changes in healthy animals. We believe that such data is essential to analyze the results obtained in sick animals.

We have also shown that a plateau in echocardiographic parameters was obtained in most animals after 4 minutes of a constant infusion rate, some animals needing up to 6 minutes. Investigators using dobutamine stimulation should be aware of these delays to avoid underestimations of the effects of the drug on the left ventricle. This is of critical importance especially if left ventricular dimensions are used to calculate fractional shortening for the evaluation of contractile reserve.

### Study limitations

The results of this study apply only to normal young adult male Wistar rats. The response of females, youngsters, older rats or other rodents to DSE needs to be assessed. Contractile reserve can be easily assessed in our protocol using a dose of 10 μg/kg/min of dobutamine as shown by the significant increase in wall thickening and ejection fraction but higher doses may need to be used to detect ischemia in animal models of ischemic heart disease.

## Conclusion

Dobutamine stress echocardiography can be easily performed in rats and can be used to evaluate left ventricular function in response to adrenergic stress. Sufficient time should be allowed for the echographic parameters to stabilize before any echographic measurements are made during dobutamine stimulation in the rat. Investigators using DSE should be aware of the normal response of the control group of their animal model to this type of stress testing.
